# A Single Intraperitoneal Secreted Protein Acidic and Rich in Cysteine Injection in Mice Is Towards an Exercise-like Phenotype

**DOI:** 10.3390/biology14040398

**Published:** 2025-04-10

**Authors:** Abdelaziz Ghanemi, Mayumi Yoshioka, Jonny St-Amand

**Affiliations:** 1 Functional Genomics Laboratory, Endocrinology and Nephrology Axis, CHU de Québec-Université Laval Research Center, Quebec, QC G1V 4G2, Canada; abdelaziz.ghanemi.1@ulaval.ca (A.G.); mayumi.yoshioka@crchudequebec.ulaval.ca (M.Y.); 2 Faculty of Pharmacy, Laval University, Quebec, QC G1V 0A6, Canada; 3 Université Laval’s Research Centre: The Tissue Engineering Laboratory (LOEX), Regenerative Medicine Division, CHU de Québec-Université Laval Research Center, Quebec, QC G1J 1Z4, Canada; 4 Department of Molecular Medicine, Faculty of Medicine, Laval University, Quebec, QC G1V 0A6, Canada

**Keywords:** secreted protein acidic and rich in cysteine (SPARC), injection, acute effect, exercise, aging, metabolism

## Abstract

Secreted protein acidic and rich in cysteine (SPARC) is a protein widely expressed in various tissues. The metabolic and functional exploration of SPARC indicated it as a mediator of the exercise-induced effects. Furthermore, SPARC overexpression mimics exercise effects (including anti-aging phenotype), whereas its knockout both reduces the exercise-induced phenotype and increases aging. Each of these previous studies has been carried out for weeks and, therefore, indicates chronic effects of SPARC. To complete the puzzle, there is a need to explore the acute effects of SPARC. Thus, this study reports results of selected molecular and metabolic explorations of mice following a single injection of SPARC. Following both a validation of the Western blot as a detection method of SPARC in the serum and the optimization of the post-injection sacrifice time, mice (male and female) were injected with either SPARC or saline and sacrificed after 4 h. Body weight, selected tissues weights, and glycemia were measured. Muscle (tibialis anterior)—that was also harvested after the sacrifice and frozen—was used to measure the expression of selected proteins related to metabolism, protein hemostasis, and muscle development. Briefly, the results indicate a protein expression pattern towards improved glucose metabolism, oxidative phosphorylation, mitochondrial biogenesis, extracellular matrix remodeling, myogenesis, and protein synthesis. On the other hand, the expression of other proteins is towards decreased muscle protein degradation. There were no significant effects of SPARC injection on glycemia. These findings represent an important step towards developing a pharmacology based on injecting SPARC to achieve therapeutic effects that basically mimic exercise benefits, including anti-aging, metabolic enhancement, and muscle development. This is of particular importance for individuals who are unable to perform the required physical activity due to physical disabilities, aging, or hospitalization.

## Simple Summary

Many individuals with health problems or conditions (disabilities, aging, etc.) are not able to perform the physical activity they need as therapy. In this context, the molecule SPARC, which is produced by muscles during exercise, has been pointed out by previous studies as producing/mediating the effects of exercise (such as metabolic benefits). In this study, we injected SPARC into mice and found that it improves the metabolic functions in the muscles (mainly glucose and proteins). These results highlight SPARC as a potential therapeutic agent that would produce exercise-like effects, therefore, providing options for individuals who need exercise benefits but are unable to perform the required physical activity.

## 1. Introduction

Studying benefits related to physiological changes in response to lifestyle (activity, diet, sleep, etc.) modifications has led to different therapeutic approaches, either directory (such as dietary restrictions) or indirectly (pharmacology) derived from observing the lifestyle-induced changes. Exercise has many well-documented effects because of which have made it an important therapeutic tool due to its beneficial effects at many levels. A challenge arises with individuals who are not able to perform the required physical activity because of physical disabilities, aging, or hospitalization. Therefore, pharmacological options must be considered.

Within this context, and among what biomedical research has pointed to, we have SPARC that has interesting exercise-like properties and was characterized as an exercise-induced gene in vivo [1]. We have shown that exercise effects are most probably, at least in part, mediated by SPARC [2], and that transgenic mice overexpressing SPARC have an exercise-like phenotype (increased muscle strength, muscle mass, and expressions of the muscle glucose transporter and mitochondrial oxidative phosphorylation, as well as decreasing glycemia and adiposity) with anti-aging patterns among other benefits [3].

It is worth noting that SPARC expression is mostly related to cell turnover and tissular modifications. Indeed, SPARC, also known as basement membrane-40 or osteonectin [4,5], is a glycoprotein with three distinct structural domains [6] and binds to calcium, collagen [7], and vitronectin (structural matrix proteins) [8]. SPARC is involved in tissue repair [9], cell turnover [10], cell renewal and growth [11,12], maintenance of bone mass [13], osteoblast maturation [4], angiogenesis regulation [13], extracellular matrix (ECM) organization [14], collagen maturation [15], glucose and lipid metabolism [16,17,18], remodeling [19,20], regeneration [21], differentiation [22], and adipose tissue regulation [23]. In addition to in vivo studies, in vitro studies showed that the electrical pulse stimulation applied to myotubes (considered as an in vitro model of exercise) also leads to *Sparc* overexpression [24] with mechanisms shared with molecular changes of exercise following the addition of SPARC to muscle cells (mitochondrial proteins expression) [24,25].

The literature highlighted above presents SPARC as a possible exercise substitute [26]. Therefore, the idea of therapeutically exploiting SPARC to obtain exercise-like effects comes out as an approach to overcome the challenges of the inability to perform exercise or to provide pharmacological tools to optimize the existing therapies. Such a pharmacological possibility requires further understanding of SPARC effects. So far, we have data on “chronic effects” through SPARC overexpressed for long durations either by exercise or by genetic modifications (transgenic mice overexpressing SPARC). Thus, additional results have to be collected regarding “acute effects” of SPARC for a full understanding of SPARC-related mechanistic effects. Indeed, among the remaining questions is whether acute effects would be similar to chronic effects, or whether they would have specific patterns in terms of intensity or the targeted pathways.

The aim of this study is to take an additional step towards such a potential therapeutic application. To contribute to filling gaps in the current literature, we evaluate selected molecular and metabolic effects of an intraperitoneal injection of a recombinant SPARC (rSPARC) in male and female mice. The aim is to have data on” acute effects” of SPARC, as only one injection is given prior to proceeding with measures rather than weeks of exercise or using mice that are overexpressing SPARC, which both reflect the “chronic effects” of SPARC and how these effects would contribute to SPARC-related potential therapeutic applications.

## 2. Materials and Methods

Briefly, the design consists of exploring selected acute effects at the molecular level following a single injection of SPARC.

Our work is divided into two steps. First, we determine the timepoint corresponding to the maximum acute effect of SPARC following SPARC injection. We select that timepoint as the one at which the serum rSPARC level reaches its maximum following SPARC injection. Once that time point is determined, it will correspond to the time at which mice will be sacrificed for our measures.

The same mice were used for both steps. The C57BL/6J mice (6 male and 6 female) were purchased from the Jackson Lab (https://www.jax.org/, accessed on 1 December 2024) at 7 weeks old and kept at our animal facility throughout the experimental steps until the date of sacrifice. The mice we used have the same genetic background as those we previously used (C57BL/6J) to study both SPARC knockout mice and mice overexpressing SPARC. C57BL/6J mice have a lifespan of around 104 weeks (26 months) [27,28].

Furthermore, mice of this study—similarly to those of our previous studies—were all housed in the same animal facility of the CHU de Québec-Université Laval Research Center (12 h light/dark cycle), under the same conditions, fed with the same chow diet (Teklad global 18% protein rodent diets [29]), had access to food and water ad libitum during the whole experimental period (except for fasting periods during which they had access to water only). All mice were sacrificed by decapitation following isoflurane inhalation anesthesia.

### 2.1. Validation of Western Blot Method to Measure the Injected rSPARC in Serum

The next step (Section 2.2) requires using a method to quantify rSPARC in the serum. Therefore, we first validated the Western blot conditions to be used. Following optimization, the protocol detailed in the next paragraph was validated. We used a pool of three sera from three young male mice (C57BL/6J) that were not exercised (from our previous study [2]), and rSPARC (10, 20, and 40 ng) was directly added to the serum (1 in 20 dilution). As a reference, lysate (3 µg protein) from the tibialis anterior (TA) muscle of a *Sparc* transgenic mouse (from another previous study [3]) was used (muscle SPARC). We had a total of 9 wells in our Western blot gel: serum (2), serum + 10 ng of rSPARC (2), serum + 20 ng of rSPARC (2), serum + 40 ng of rSPARC (2), and TA muscle lysate (3 µg protein). rSPARC (AF942-SP, Recombinant Mouse SPARC, CF, 50 µg) was purchased from R&D Systems Inc. (Minneapolis, MN, USA).

Proteins were separated by sodium dodecyl sulfate polyacrylamide gel electrophoresis (SDS-PAGE) using the TGX Stain-Free FastCast acrylamide solutions (Bio-Rad Laboratories Ltd., Mississauga, ON, Canada), and the trihalo compound in the gels was activated under UV light. Then, total proteins were transferred at room temperature to polyvinylidene fluoride (PVDF) membranes (Bio-Rad Laboratories Ltd.) with the BioRad Trans-Blot Turbo Transfer System (1.5 mm). Gels (before and after the transfer) and membranes were visualized under UV light by using the AlphaImager TM 1220 (Alpha Innotech Co., San Leandro, CA, USA). Membranes were blocked using the Pierce™ Protein-Free (TBS) blocking buffer (Life Technologies Inc., Burlington, ON, Canada), incubated overnight with 1/2000 dilution (in the blocking buffer) of primary antibodies (AF942-SP, R&D Systems Inc., Minneapolis, MN, USA) and 2 h incubation with 1/1000 dilution (in the blocking buffer) of secondary antibodies (sc-2354, Santa Cruz Biotechnology Inc., Dallas, TX, USA), and finally visualized with the Clarity™ Western ECL Blotting Substrate on a film (1705060, Bio-Rad Laboratories Ltd.) and/or using Fusion Fx7 (Montreal Biotech Inc., Montreal, QC, Canada). First, the visualized total proteins on the membranes and target proteins on the films were quantified using ImageJ software version 1.52e (ImageJ bundled with 64-bit Java 1.8.0_172, U. S. National Institutes of Health, Bethesda, MD, USA) [30]. The methodology of lane and band quantifications, followed by expression evaluations, was performed according to Taylor et al. [31,32] as we have detailed in one of our previous work [33].

### 2.2. Finding How Long Following Intraperitoneal rSPARC Injection Does It Take (T Max) to Reach the Maximum Serum Concentration

The C57BL/6J mice used for this study were purchased at the age of 7 weeks from the Jackson Laboratory (https://www.jax.org/, accessed on 1 December 2024) and kept at our animal facility until the day of sacrifice (Section 2.3).

This step was performed when mice were between 12 and 13 weeks old. Each mouse has been given one intraperitoneal injection (intraperitoneal injection of SPRAC was already reported in the literature [34]) and based on the paper of Aoi et al., in which they studied the exercise-induced SPARC secretion from muscles that inhibits tumorigenesis [35]. Blood (20 µL) was collected through the tail vein at pre (0 h) and post (2 h, 4 h, and 8 h) injections. Circulating SPARC levels were measured by Western blot (same conditions and steps as in the previous section, Section 2.1) to determine the time required to reach the Tmax. For each mouse, the test (injection followed by rSPARC serum level quantification) was carried out twice, with a 1-week interval to have two different tests/measures of Tmax.

### 2.3. Experimental Design and rSPARC Injection to Mice

Following the determination of T max (4 h, please see the section results), and to keep the same conditions used during the Tmax determination (2.2.), the same mice used to determine T max were used for the remaining parts of the study. However, the period between step 2.2 (mice age: 12 to 13 weeks) and this step (2.3) was 4 to 6 weeks (mice age: 17 to 18 weeks), so that the effects of the previous injections (used to determine Tmax) have no impact on the subsequent measures. In addition, the mice injected with saline at this step have also been used during step 2.2. Therefore, all the mice injected with rSPARC or saline at the age of 17 to 18 weeks have previously been injected with rSPARC when they were 12 to 13 weeks old. This allows several weeks of differences for the clearance of the injected rSPARC (which, as we found below, reaches its peak and starts to drop just 4 h post-injection).

Such an approach is important as it further optimizes our design, as we would have used the same mice for both determining the Tmax and conducting our evaluation of post-injection molecular effects.

We had a total of 12 mice (6 males and 6 females, age: 17 to 18 weeks), for each sex, 3 mice were injected with rSPARC (30 µg rSPARC/kg body weight with a concentration of 5 ng rSPARC per µL saline) and 3 were injected with saline (6 mL/g body weight). Therefore, we have four experimental conditions: male mice injected with rSPARC, male mice injected with saline, female mice injected with rSPARC, and female mice injected with saline.

Once the optimum time, corresponding to the pic of the rSPARC serum concentration, was determined as 4 h, we proceeded with the exploration of rSPARC injection effects by sacrificing the mice 4 h post-injection.

Between the ages of 17 and 18 weeks, mice were sacrificed 4 h (T max) after saline or rSPARC intraperitoneal injection (given at the same amount and concentration used to determine Tmax in Section 2.1). At Tmax, mice were anesthetized (isoflurane inhalation) and euthanized by decapitation, blood collected, and tissues harvested.

#### 2.3.1. Blood Glucose (Glycemia)

Prior to the injections (rSPARC or saline), mice were fasted for 12 h. Following the sacrifice (4 h post-rSPARC or saline injection), blood was collected, and blood glucose levels were measured using glucose test strips that were then inserted into a blood glucose meter (Accu-Chek, Roche Diabetes Care, Inc., Mississauga, ON, Canada) to read the blood glucose values.

#### 2.3.2. Tissues’ Weights

After the mice sacrifice, the following tissues were harvested and weighed: liver, heart, retroperitoneal adipose tissue, inguinal adipose tissue, paraovary adipose tissue (females) and epididymal adipose tissue (males), and skeletal muscles (soleus, gastrocnemius, and TA).

For the TA muscle, used later for Western blot, it was snap frozen in liquid nitrogen, then moved to −80 °C, and stored until the protein extraction.

#### 2.3.3. Muscle Proteins Expression (Western Blot)

To explore selected metabolic, biochemical, and functional indicators, we measured the post-injection acute changes of the expression of selected proteins in TA with Western blot. We measured the expression of the proteins in TA muscle because it is the skeletal muscle for which we had an effect of age (decrease), genotype (decrease), and exercise (increase) on either its weight or weight percentage in our previous SPARC study [2]. Thus, it is more likely to be sensitive to rSPARC injection effects.

The proteins for which the muscular protein was measured were mitochondrial cytochrome c oxidase 2 (COX2), collagen type I alpha 2 chain, (COL1A2), eukaryotic translation initiation factor 4E-binding protein 1 (4E-BP1), F-box protein 32/atrogin (Fbx32/Atrogin 1), glucose transporter 4 (GLUT4), glycogen synthase kinase 3 beta (GSK-3β), interleukin 6 (IL6), integrin-linked kinase (ILK), myogenic differentiation 1 (MyoD1), Myogenin, nuclear respiratory factor 1 (NRF-1), phosphorylated (Ser65) eukaryotic translation initiation factor 4E (eIF4E)-binding protein 1 (p4E-BP1), phosphorylated (Ser473) Ak strain transforming/protein kinase B (pAkt1/PKB), phosphorylated (α1-Thr183 and α2-Thr172) AMP-activated protein kinase (pAMPK), phosphorylated (Ser256) forkhead box protein O1 (pFoxO1), peroxisome proliferator-activated receptor gamma coactivator-1α (PGC1α), phosphorylated (Ser-9) glycogen synthase kinase 3 beta (pGSK-3β), phosphorylated (Thr389) ribosomal protein S6 kinase beta-1 (pS6K1), phosphorylated (Ser423/Ser425) SMAD family member 3 (pSmad3), succinate dehydrogenase [ubiquinone] iron-sulfur subunit, mitochondrial (SDHB), and muscle ring-finger protein-1 (TRIM63/MuRF1).

To measure the expression of these selected proteins, the total proteins were extracted from the TA muscle, using a radio-immunoprecipitation assay (RIPA) buffer and a protease inhibitor cocktail (Sigma-Aldrich Canada Co., Oakville, ON, Canada), and followed by a protein quantification of each protein extract using a Bio-Rad protein assay (Bio-Rad Laboratories Ltd.). The protein extracts were kept at −80 °C until the Western blot was performed.

Western blot of the proteins was performed following the same steps as for SPARC (Section 2.1) with specific conditions for each protein. The Western blot conditions of these proteins (as those of SPARC) were shown in Supplementary Material S1: The loaded protein amounts, used gels, transfer, blocking, primary antibody incubation, secondary antibody incubation, and the visualization conditions.

#### 2.3.4. Statistical Analyses

The data were analyzed by 2-way ANOVA (rSPARC injection and sex). When a significant (*p* ≤ 0.05) or trend (0.05 < *p* ≤ 0.1) in interaction was found, the rSPARC effect in males or the rSPARC effect in females was identified using a post-hoc test (mean comparison). We have also performed further statistical analyses (including effect size and additional T tests validations).

## 3. Results

### 3.1. Validation of rSPARC Detection by Western Blot

**Figure 1:**
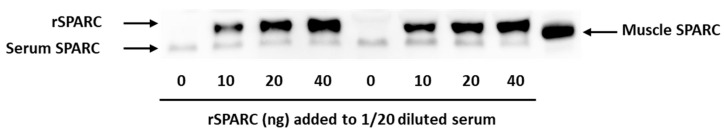
Detection of rSPARC by Western blotting.

As shown in Figure 1, the Western blot conditions (Section 2.1 and Supplementary Material S1) have been validated to detect rSPARC in mouse serum. rSPARC blots increase according to the loaded amount (0, 10, 20, and 40 ng).

The increase in rSPARC in the serum was detected by Western blot, when rSPARC (10, 20, and 40 ng) was directly added to the serum (1 in 20 dilution) from C57BL/6J. As a reference, lysate (3 µg protein) from TA muscle of a *Sparc* transgenic mouse was used (muscle SPARC).

**Abbreviations: SPARC**: secreted protein acidic and cysteine rich; **rSPARC**: recombinant mouse SPARC.

### 3.2. Determination of the Optimum Timepoint Tmax

**Figure 2:**
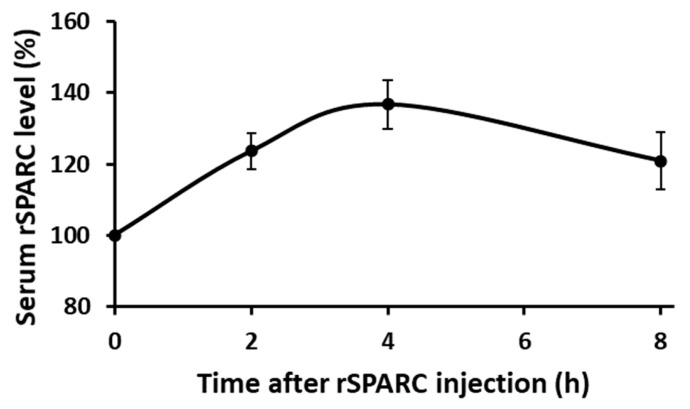
Serum rSPARC levels after rSPARC injection in mice.

Following the validation of Western blot conditions allowing for to quantification of serum rSPARC level, we determined the Tmax as the time required to reach the maximum rSPARC serum levels after rSPARC intraperitoneal injection for each mouse. As shown in Figure 2, the Tmax is 4 h. More technical details on the determination of T max by Western blotting can be found in Supplementary Material S2.

The mice (C57BL/6J: 6 males and 6 females) were purchased from the Jackson Laboratory. At the age of 11 or 12 weeks, rSPARC (30 µg rSPARC/kg body weight with a concentration of 5 ng rSPARC per µL saline) was injected intraperitoneally, and blood (20 µL) was collected through the tail vein at pre (0 h) and post (2 h, 4 h, and 8 h) injections. Circulating SPARC levels were measured by Western blot to determine the time to reach maximum concentration (T max).

**Abbreviations: rSPARC**: recombinant mouse SPARC.

### 3.3. Post-Injection Analyses

Our experimental design had four groups (males injected with saline, males injected with rSPARC, females injected with saline, and females injected with rSPARC). The mice were injected with rSPARC or saline and sacrificed after 4 h (Tmax, previously determined).

#### 3.3.1. Body Weights and Tissues Weights and Injection Volumes (Table 1)

We had no rSPARC injection effects on the body weights (thus, the injected volumes), tissues’ weights, or glycemia.

**Table 1:** Body weight, injection volume, blood glucose, and organ/tissue weight.

			Male						Female							2-way ANOVA						
			Saline			rSPARC				Saline			rSPARC			*p* Value		Effect Size (h^2^)				
																Treatment	Sex	Interaction	Treatment	Sex	Interaction	
**Body weight (g)**			31.7	±	2.0	31.2	±	1.3		22.9	±	1.3	23.4	±	2.7	0.99	0.0001	0.65	0.00	0.88	0.03	
**Injection volume (mL)**			190	±	12	187	±	8		137	±	8	140	±	17	1.00	0.0001	0.67	0.00	0.87	0.02	
**Blood glucose (mM)**			9.7	±	0.5	9.4	±	2.3		6.0	±	1.2	6.2	±	0.6	0.97	0.002	0.75	0.00	0.70	0.01	
**Organ/Tissue weight (mg)**																						
	**Liver**		1.14	±	0.09	1.05	±	0.15		0.75	±	0.07	0.82	±	0.09	0.88	0.001	0.21	0.00	0.77	0.19	
	**Heart**		0.133	±	0.012	0.146	±	0.011		0.109	±	0.020	0.100	±	0.002	0.87	0.001	0.17	0.00	0.74	0.22	
	**Adipose tissue**																					
		**Retroperi-toneal**	0.235	±	0.076	0.139	±	0.035		0.056	±	0.040	0.101	±	0.097	0.52	0.02	0.11	0.05	0.49	0.29	
		**Inguinal**	0.364	±	0.090	0.295	±	0.046		0.215	±	0.085	0.288	±	0.130	0.97	0.18	0.22	0.00	0.21	0.18	
		**Para-ovary**								0.814	±	1.146	0.967	±	0.913	0.87 *	-	-	0.15 **	-	-	
		**Epidi-dymal**	0.838	±	0.198	0.616	±	0.103								0.16 *	-	-	1.41 **	-	-	
	**Skeletal muscle**																					
		**Soleus**	0.033	±	0.010	0.034	±	0.009		0.021	±	0.006	0.024	±	0.008	0.76	0.05	0.90	0.01	0.39	0.00	
		**Gastro-cnemius**	0.334	±	0.023	0.330	±	0.040		0.244	±	0.010	0.233	±	0.036	0.67	0.001	0.85	0.02	0.79	0.00	
		**Tibialis anterior**	0.120	±	0.018	0.130	±	0.012		0.082	±	0.004	0.084	±	0.003	0.40	0.0002	0.56	0.09	0.85	0.04	

Data are normalized with protein quantity on each lane of the membrane and shown as mean ± SD (N = 3 for male or female and N = 6 for all). * *T*-test. ** dCohen effect size. Significant effect: *p* ≤ 0.05; Trend: 0.05 < *p* ≤ 1.0. Large effect: η^2^ ≥ 0.14; Medium effect: 0.06 ≤ η2 < 0.14. Large effect: d ≥ 0.8; Medium effect: 0.5 ≤ d < 0.8.

#### 3.3.2. Western Blot Analyses (Table 2)

The protein expression has shown various patterns. The figures of the protein expression (bar graphs) have been inserted within the graphical conclusion in the discussion (Section 4).

For sex effects, the expression of 4E-BP1, p4E-BP1, pAMPK, COL1A2 (trend), Fbx32 (trend), ILK, myogenin, and PGC1α is higher in females, whereas the expression of pAkt1 is higher in males.

The rSPARC injection increases the expressions of pAkt1, pAMPK, COX2 (trend), GLUT4 (trend), pGSK3β, ILK, myogenin, and PGC1α and decreases the expressions of pFoxO1, pSmad3, and Fbx32.

Regarding the interactions (rSPARC effect in males or rSPARC effect in females), we found that using a post-hoc test (mean comparison), in males, rSPARC injection increases the expressions of COL1A2, pGSK3β, and NRF1. In females, rSPARC injection increases the expression of pAMPK and myogenin but decreases the expression of 4E-BP1, COL1A2 (trend), and Fbx32.

For the expression of GSK3β, IL6, MyoD1, pS6K1, SDHB, and TRIM63, no effects (nor trends) have been found for any of the two variables (rSPARC injection and sex).

As for the normalization of the Western blot, we have used the total protein after transfer that was quantified, and after that, each target protein blot quantification was divided by the corresponding lane of the total protein after transfer. Supplementary Material S3 shows all the images of both total proteins after the transfer and target protein blots.

**Table 2:** Western blot results.

	Male						Female						2-Way ANOVA									
	Saline			rSPARC			Saline			rSPARC			*p* Value					Effect Size (h^2^)			d_Cohen_ Effect Size	
													Treatment	Sex	Interaction	Male	Female	Treatment	Sex	Interaction	Male	Female
**ILK**	0.52	±	0.05	0.99	±	0.24	1.09	±	0.13	1.32	±	0.14	0.01	0.003	0.31	-	-	0.56	0.68	0.13	-	-
**GSK-3b**	0.95	±	0.11	0.87	±	0.04	1.04	±	0.27	1.13	±	0.33	0.99	0.28	0.61	-	-	0.00	0.14	0.03	-	-
**pGSK-3b**	0.95	±	0.11	0.87	±	0.04	1.04	±	0.27	1.13	±	0.33	0.02	0.70	0.05	0.01	0.69	0.51	0.02	0.40	1.0	0.3
**COL1A2**	0.41	±	0.17	1.11	±	0.24	1.50	±	0.46	0.83	±	0.29	0.95	0.10	0.01	0.05	0.06	0.00	0.30	0.55	3.4	1.7
**pAkt1**	0.79	±	0.33	2.21	±	0.86	0.33	±	0.05	0.66	±	0.16	0.03	0.02	0.14	-	-	0.46	0.53	0.25	-	-
**pS6K**	1.15	±	0.54	0.88	±	0.43	0.94	±	0.27	1.08	±	0.20	0.82	1.00	0.46	-	-	0.01	0.00	0.07	-	-
**p4EBP1**	0.53	±	0.08	0.75	±	0.34	1.10	±	0.25	1.40	±	0.34	0.22	0.01	0.83	-	-	0.18	0.55	0.01	-	-
**4EBP1**	0.59	±	0.28	1.08	±	0.31	1.48	±	0.10	0.74	±	0.41	0.58	0.23	0.02	0.14	0.04	0.04	0.17	0.51	1.7	2.5
**IL6**	1.14	±	0.13	1.02	±	0.19	0.89	±	0.01	0.98	±	0.05	0.85	0.12	0.23	-	-	0.00	0.28	0.17	-	-
**pAMPK**	0.45	±	0.10	0.43	±	0.16	1.01	±	0.05	1.92	±	0.41	0.0002	0.02	0.02	0.93	0.004	0.49	0.84	0.51	0.1	3.1
**GLUT4**	0.98	±	0.11	1.39	±	0.47	0.66	±	0.09	1.04	±	0.18	0.11	0.07	0.93	-	-	0.36	0.29	0.00	-	-
**PGC1a**	0.24	±	0.12	0.81	±	0.21	1.11	±	0.22	1.64	±	0.19	0.003	0.0002	0.89	-	-	0.68	0.83	0.00	-	-
**NRF1**	0.64	±	0.23	1.32	±	0.44	1.15	±	0.10	0.91	±	0.32	0.33	0.82	0.06	0.05	0.43	0.12	0.01	0.37	1.9	0.3
**COX2**	0.87	±	0.22	1.17	±	0.10	0.91	±	0.18	1.06	±	0.01	0.07	0.72	0.50	-	-	0.36	0.02	0.06	-	-
**SDHB**	0.90	±	0.36	1.11	±	0.21	1.02	±	0.10	0.98	±	0.17	0.63	0.97	0.44	-	-	0.03	0.00	0.07	-	-
**pSmad3**	1.21	±	0.07	0.90	±	0.06	0.99	±	0.16	0.91	±	0.05	0.02	0.14	0.14	-	-	0.50	0.25	0.25	-	-
**pFOXO1**	1.33	±	0.61	0.48	±	0.19	1.34	±	0.50	0.81	±	0.13	0.04	0.58	0.59	-	-	0.41	0.04	0.04	-	-
**TRIM63**	1.49	±	0.82	0.83	±	0.38	0.82	±	0.17	0.89	±	0.32	0.42	0.39	0.32	-	-	0.08	0.09	0.12	-	-
**Fbx32**	0.65	±	0.28	0.71	±	0.06	1.54	±	0.27	1.02	±	0.23	0.19	0.01	0.10	0.80	0.05	0.20	0.63	0.29	0.3	2.1
**Myod1**	1.04	±	0.24	0.93	±	0.12	0.94	±	0.07	1.09	±	0.18	0.90	0.81	0.30	-	-	0.00	0.01	0.13	-	-
**Myogenin**	0.21	±	0.15	0.36	±	0.15	1.12	±	0.53	2.32	±	0.23	0.01	0.0002	0.04	0.63	0.005	0.55	0.85	0.42	1.0	2.9

Data are normalized with protein quantity on each lane of the membrane and shown as mean ± SD (N = 3 per group). Significant effect: *p* ≤ 0.05; Trend: 0.05 < *p* ≤ 1.0. Large effect: η^2^ ≥ 0.14; Medium effect: 0.06 ≤ η2 < 0.14. Large effect: d ≥ 0.8; Medium effect: 0.5 ≤ d < 0.8. **Abbreviations**: **COX2**: mitochondrial cytochrome c oxidase 2; **COL1A2**: collagen type I alpha 2 chain; **Fbx32**: F-box protein 32/Atrogin 1; **GLUT4**: glucose transporter 4; **IL6**: interleukin 6; **ILK**: integrin-linked kinase; **Myod1**: myogenic differentiation 1; **NRF1**: regulating nuclear respiratory factor 1; **p4EBP1**: phosphorylated (Ser65) eukaryotic translation initiation factor 4E (eIF4E)-binding protein 1; **pAkt**: phosphorylated (Ser473) Ak strain transforming/protein kinase B; **pAMPK**: phosphorylated (α1-Thr183 and α2-Thr172) adenosine monophosphate-activated protein kinase; **pFoxO1**: phosphorylated (Ser256) forkhead box protein O1; **PGC1α**: peroxisome proliferator-activated receptor gamma coactivator-1α; pGSK-3b: phosphorylated (Ser-9) glycogen synthase kinase 3 beta; **pS6K1**: phosphorylated (Thr389) ribosomal protein S6 kinase beta-1; **pSmad3**: phosphorylated (Ser423/Ser425) SMAD family member 3; **SDHB**: succinate dehydrogenase [ubiquinone] iron-sulfur subunit, mitochondrial; **rSPARC**: recombinant protein secreted protein acidic and rich in cysteine; **TGF-β1**: transforming growth factor beta 1; **TRIM63**: tripartite motif-containing 63/muscle-specific RING finger protein 1.

## 4. Discussion

Following the validation of Western blot conditions to quantify rSPARC in the serum following rSPARC intraperitoneal injection, we determined the time corresponding to the highest concentration following a single rSPARC injection (Tmax) as 4 h. Tmax has been the post-injection time points for the subsequent study that consisted of exploring molecular and metabolic effects of rSPARC injection. Following the injections, mice had no local reactions or side effects.

As SPARC serum levels increase with exercise [36] both in humans and mice [35], the rationale of the study lies in SPARC injection leading to an increase in SPARC serum levels. Thus, mimicking the exercise-induced SPARC serum level increase, as our hypothesis, is that SPARC mediates, at least a part of, the exercise-induced benefits at the molecular, biochemical, and functional levels [2].

The absence of any significant difference in body and tissues’ weights between the mice injected with rSPARC and those injected with saline further validates the choice of the mice and highlights that the differences observed for the proteins expression in the muscle results from rSPARC injection rather than from a pre-existing differences in tissues size leading to an increased metabolic or biochemical activity or to a difference in the internal bioenvironmental conditions that could impact the signaling and the production of hormones or other cytokines impacting the metabolism and the muscle development. Furthermore, the absence of statistically significant differences in terms of body weights between groups resulted in the absence of any statistical difference in the injected volumes between groups, except for sex differences. Therefore, excluding pharmacokinetic factors related to the distribution volumes that could impact the pharmacodynamics of an injected molecule as well as the pain sensation [37] and adverse events [38] at the injection site that could stimulate pathways related to our measures. In addition, although chronic SPARC exposure or exercise would typically affect body weights and tissues’ weights, it is normal (even if we had a SPARC-inducing exercise level) not to see any changes in body weights and tissues’ weights within 4 h, as such changes in weights would require much more time.

Exercise is known to promote muscle growth and mitochondrial biogenesis via activation of IGF1-PI3K-Akt-mTOR [39,40,41,42,43,44,45,46,47] and AMPK-PGC1α [48,49,50] pathways, respectively, as well as via inhibition of protein degradation (FoxO and Smad3 pathways [51,52,53,54]). Exercise-induced gene, SPARC, regulates ECM remodeling via ILK-GSK 3β-β catenin [55,56] and TGF β1-Smad3 pathways [57]. Although inactivation of the *Smad3* gene in mice paradoxically shows accelerated wound healing [58], SPARC binds to TGF-β1 co-receptor and inhibits the binding of TGF-β1 to its receptor [59]. Thus, the TGF β1-Smad3-atrogin 1 pathway suppresses myogenic transcription factors (Myo D and myogenin) degradation and promotes muscle differentiation [60]. SPARC also interacts with AMPK [24,61], which induces PGC1α [48] and stimulates ILK-GSK 3β-PGC1α pathway [62], which may lead to mitochondrial biogenesis through a powerful induction of NRF1 [50].

Injection of rSPARC, on the other hand, in mice activated pathways of ECM remodeling (ILK-GSK 3β-COL1A2), glucose metabolism (AMPK-GLUT4), and mitochondrial biogenesis (AMPK-PGC1α-NRF1-COX2 and ILK-GSK 3β-PGC1α-NRF1-COX2) in the skeletal muscle. It also suppressed the pathway of muscle protein degradation (Akt1-Smad3/FoxO1-Atrogin 1-Myogenin), which may, in turn, activate myogenic differentiation. On the other hand, for the pathways of protein synthesis, the rSPARC-induced decrease of 4E-BP1 would indicate a phosphorylation (conversion of 4E-BP1 into p4E-BP1) in the context of protein synthesis activation [63]. The graphical conclusion below compiles the findings into a set of hypothetical mechanistic pathways.

In addition, sex differences and/or interaction between rSPARC and sex were found in several pathways such as ECM remodeling, mitochondrial biogenesis, muscle protein degradation, and myogenesis (Graphical conclusion).

Importantly, to illustrate the similarities between the consequences of rSPARC injection (our results) and exercise benefits, we point out that exercise increases the expression of GLUT4 [64], COX2 [65], NRF1 [65], PGC1α [66], ILK [67], pGSK-3β [68], and myogenin [69] protein or gene expression, whereas it reduces the expression of both TGF-β1 and pSmad3 protein or gene expression [70]. Furthermore, SPARC enhances glucose uptake (and glucose tolerance) in skeletal muscles [71] and adding or inducing SPARC in C2C12 muscle cells increased their differentiation, myogenin expression, collagen expression, and the expression of two mitochondrial proteins (including SDHB [25] and PGC-1α [24]). This further points to SPARC as the exercise mediator leading to important outcomes of the resulting phenotype, which is similar to our results.




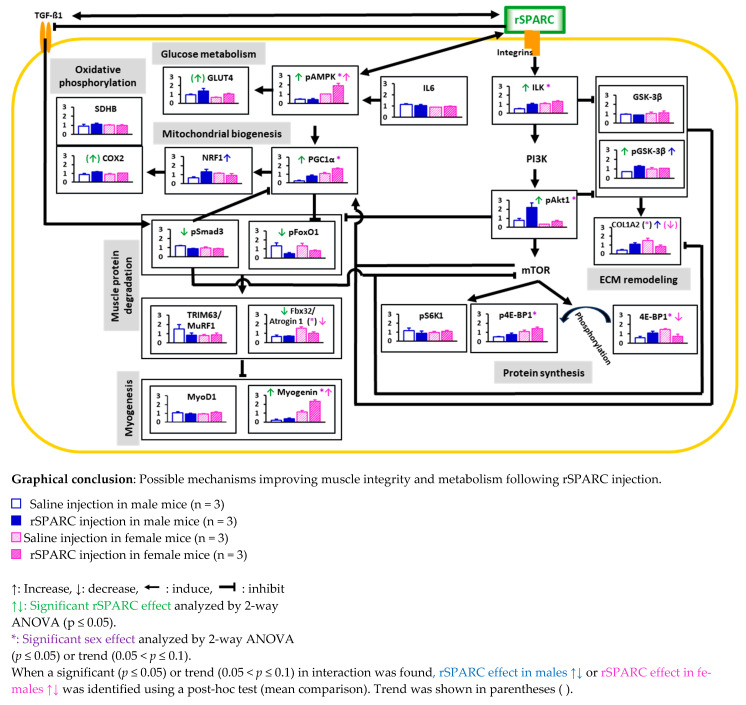

**Abbreviations: COL1A2**: collagen type I alpha 2 chain; **COX2**: mitochondrial cytochrome c oxidase 2; **ECM**: extracellular matrix; **4E-BP1**: eukaryotic translation initiation factor 4E-binding protein 1; **Fbx32/Atrogin 1**: F-box protein 32/atrogin 1; **GLUT4**: glucose transporter 4; **GSK-3β**: glycogen synthase kinase 3 beta; **IL6**: interleukin 6; **ILK**: integrin-linked kinase; **mTOR**: mammalian target of rapamycin; **MyoD1**: myogenic differentiation 1; **NRF-1**: nuclear respiratory factor 1; **p4E-BP1**: phosphorylated (Ser65) eukaryotic translation initiation factor 4E (eIF4E)-binding protein 1; **pAkt1/PKB**: phosphorylated (Ser473) Ak strain transforming/protein kinase B; **pAMPK**: phosphorylated (α1-Thr183 and α2-Thr172) AMP-activated protein kinase; **pFoxO1**: phosphorylated (Ser256) forkhead box protein O1; **PGC1α**: peroxisome proliferator-activated receptor gamma coactivator-1α; **PI3K**: phosphoinositide 3; **pGSK-3β:** phosphorylated (Ser-9) glycogen synthase kinase 3 beta; **PI3K**: phosphoinositide 3; **pS6K1**: phosphorylated (Thr389) ribosomal protein S6 kinase beta-1; **pSmad3**: phosphorylated (Ser423/Ser425) SMAD family member 3; **SDHB**: succinate dehydrogenase [ubiquinone] iron-sulfur subunit, mitochondrial; **rSPARC**: recombinant secreted protein acidic and rich in cysteine; **TGF-β1**: transforming growth factor beta 1; **TRIM63/MuRF1**: tripartite motif-containing 63/muscle-specific RING finger protein 1.

These findings provide direct mechanistic links between the pathways underlying a part of the acute exercise-induced phenotype. The possible practical implications deriving from such observations, including research and clinical applications, are discussed in the next section.

## 5. Conclusions

Although we have mentioned SPARC as a potential medication, the concept is not to encourage a sedentary lifestyle but rather to provide an option for those suffering from conditions preventing them from performing the required physical activity, such as physical disabilities.

Indeed, exercise remains a key approach as muscle contraction (via releasing myokines) produces beneficial effects [72,73,74] that might not be seen in other strategies that do not involve muscle activation. Muscle is a tissue directly related to movement, but when activated, it also becomes a key regulator of metabolism (among other roles).

Coming back to our data, the key finding is that our results confirm those available in the literature and make it worth exploring whether repeated SPARC injections could lead to similar effects of an overexpression of SPARC or an exercise-induced SPARC. We are also optimistic about the extent to which we could extrapolate such animal results to humans, as sequences of the *SPARC* gene and protein are highly conserved among species [10].

On the other hand, since *Sparc* expression is downregulated by aging [35] and exercise both increases *Sparc*/SPARC [35,75] and counteracts aging, SPARC injections could be optimized towards an anti-aging approach, in addition to the pharmacological benefits related to SPARC properties (metabolic booster, protein synthesis, etc.).

We have chosen to conduct this part of our investigation on both male and female mice to include the sex factor, even though skeletal muscle development, response to exercise, as well as sarcopenic (age-related) muscle mass and strength loss are more important in males compared to females [76,77]. In addition, exercise-induced SPARC levels correlate with skeletal muscle mass [78]. Therefore, we would have expected that male mice would have a better response to rSPARC (as we had sex effects for some tissues’ weights), but it was not significantly the case. This indicates that although SPARC is an important myokine, non-muscular factors are probably involved in its response and highlight once again the importance of including sex as a variable in our studies, especially as our results show that the same injection can, for instance, increase a factor in males but decrease it in females.

It is also crucial to distinguish between SPARC acute effects and chronic effects, as this will determine the frequency and the form of pharmacological usage of SPARC depending on the desired effects. Within this context, our results provide a clear pattern of similarities between rSPARC injection and exercise-like effects. However, some effects were not significant enough, probably because a single injection would not provide sufficient effects to obtain the required “therapeutic phenotype” and could require more than one injection. Therefore, further studies are required to both validate the possible side effects mainly resulting from repeated injections and explore possible routes of administration to have an alternative to injections, such as an implanted delivery system. Moreover, further mechanistic studies would provide additional steps towards the potential therapeutic usage of SPARC.

The importance of SPARC, the various roles it plays, and its wide distribution mean that its injection would affect more than one system and therefore leads to a relatively systemically distributed phenotype rather than a limited organ-specific or tissue-specific change. This means an increased pharmacovigilance but also more targeted tissues with possible positive outcomes since SPARC is an endogenous molecule that governs various hemostatic functions.

## Data Availability

Data are contained within the article and Supplementary Materials.

## Supplementary Materials

The following supporting information can be downloaded at https://www.mdpi.com/article/10.3390/biology14040398/s1, Supplementary S1–S3.

## References

[1] Riedl I., Yoshioka M., Nishida Y., Tobina T., Paradis R., Shono N., Tanaka H., St-Amand J. (2010). Regulation of skeletal muscle transcriptome in elderly men after 6 weeks of endurance training at lactate threshold intensity. Exp. Gerontol..

[2] Ghanemi A., Melouane A., Yoshioka M., St-Amand J. (2020). Exercise Training of Secreted Protein Acidic and Rich in Cysteine (Sparc) KO Mice Suggests That Exercise-Induced Muscle Phenotype Changes Are SPARC-Dependent. Appl. Sci..

[3] Ghanemi A., Melouane A., Yoshioka M., St-Amand J. (2022). Secreted Protein Acidic and Rich in Cysteine (Sparc) KO Leads to an Accelerated Ageing Phenotype Which Is Improved by Exercise Whereas SPARC Overexpression Mimics Exercise Effects in Mice. Metabolites.

[4] Delany A.M., Kalajzic I., Bradshaw A.D., Sage E.H., Canalis E. (2003). Osteonectin-null mutation compromises osteoblast formation, maturation, and survival. Endocrinology.

[5] Motamed K. (1999). SPARC (osteonectin/BM-40). Int. J. Biochem. Cell Biol..

[6] Scavelli K., Chatterjee A., Rhee D.J. (2015). Secreted Protein Acidic and Rich in Cysteine in Ocular Tissue. J. Ocul. Pharmacol. Ther. Off. J. Assoc. Ocul. Pharmacol. Ther..

[7] Sage H., Johnson C., Bornstein P. (1984). Characterization of a novel serum albumin-binding glycoprotein secreted by endothelial cells in culture. J. Biol. Chem..

[8] Brekken R.A., Sage E.H. (2000). SPARC, a matricellular protein: At the crossroads of cell-matrix. Matrix Biol. J. Int. Soc. Matrix Biol..

[9] Norose K., Clark J.I., Syed N.A., Basu A., Heber-Katz E., Sage E.H., Howe C.C. (1998). SPARC deficiency leads to early-onset cataractogenesis. Investig. Ophthalmol. Vis. Sci..

[10] Yan Q., Sage E.H. (1999). SPARC, a matricellular glycoprotein with important biological functions. J. Histochem. Cytochem. Off. J. Histochem. Soc..

[11] Zhu J., Wang L.Y., Li C.Y., Wu J.Y., Zhang Y.T., Pang K.P., Wei Y., Du L.Q., Liu M., Wu X.Y. (2020). SPARC promotes self-renewal of limbal epithelial stem cells and ocular surface restoration through JNK and p38-MAPK signaling pathways. Stem Cells.

[12] Alachkar H., Santhanam R., Maharry K., Metzeler K.H., Huang X., Kohlschmidt J., Mendler J.H., Benito J.M., Hickey C., Neviani P. (2014). SPARC promotes leukemic cell growth and predicts acute myeloid leukemia outcome. J. Clin. Investig..

[13] Delany A.M., Amling M., Priemel M., Howe C., Baron R., Canalis E. (2000). Osteopenia and decreased bone formation in osteonectin-deficient mice. J. Clin. Investig..

[14] Barker T.H., Baneyx G., Cardó-Vila M., Workman G.A., Weaver M., Menon P.M., Dedhar S., Rempel S.A., Arap W., Pasqualini R. (2005). SPARC regulates extracellular matrix organization through its modulation of integrin-linked kinase activity. J. Biol. Chem..

[15] Bradshaw A.D. (2009). The role of SPARC in extracellular matrix assembly. J. Cell Commun. Signal..

[16] Ghanemi A., Melouane A., Yoshioka M., St-Amand J. (2019). Secreted protein acidic and rich in cysteine and bioenergetics: Extracellular matrix, adipocytes remodeling and skeletal muscle metabolism. Int. J. Biochem. Cell Biol..

[17] M Onorato A., Fiore E., Bayo J., Casali C., Fernandez-Tomé M., Rodríguez M., Domínguez L., Argemi J., Hidalgo F., Favre C. (2021). SPARC inhibition accelerates NAFLD-associated hepatocellular carcinoma development by dysregulating hepatic lipid metabolism. Liver Int. Off. J. Int. Assoc. Study Liver.

[18] Song H., Ding L., Zhang S., Wang W. (2018). MiR-29 family members interact with SPARC to regulate glucose metabolism. Biochem. Biophys. Res. Commun..

[19] Ghanemi A., Yoshioka M., St-Amand J. (2020). Secreted Protein Acidic and Rich in Cysteine: Metabolic and Homeostatic Properties beyond the Extracellular Matrix Structure. Appl. Sci..

[20] McCurdy S., Baicu C.F., Heymans S., Bradshaw A.D. (2010). Cardiac extracellular matrix remodeling: Fibrillar collagens and Secreted Protein Acidic and Rich in Cysteine (SPARC). J. Mol. Cell. Cardiol..

[21] Petersson S.J., Jørgensen L.H., Andersen D.C., Nørgaard R.C., Jensen C.H., Schrøder H.D. (2013). SPARC is up-regulated during skeletal muscle regeneration and inhibits myoblast differentiation. Histol. Histopathol..

[22] Bradshaw A.D., Sage E.H. (2001). SPARC, a matricellular protein that functions in cellular differentiation and tissue response to injury. J. Clin. Investig..

[23] Kim J.S., Galvão D.A., Newton R.U., Gray E., Taaffe D.R. (2021). Exercise-induced myokines and their effect on prostate cancer. Nat. Rev. Urol..

[24] Melouane A., Yoshioka M., Kanzaki M., St-Amand J. (2019). Sparc, an EPS-induced gene, modulates the extracellular matrix and mitochondrial function via ILK/AMPK pathways in C2C12 cells. Life Sci..

[25] Melouane A., Carbonell A., Yoshioka M., Puymirat J., St-Amand J. (2018). Implication of SPARC in the modulation of the extracellular matrix and mitochondrial function in muscle cells. PLoS ONE.

[26] Ghanemi A., Yoshioka M., St-Amand J. (2022). Genetic Expression between Ageing and Exercise: Secreted Protein Acidic and Rich in Cysteine as a Potential “Exercise Substitute” Antiageing Therapy. Genes.

[27] Rowlatt C., Chesterman F.C., Sheriff M.U. (1976). Lifespan, age changes and tumour incidence in an ageing C57BL mouse colony. Lab. Anim..

[28] Kunstyr I., Leuenberger H.G. (1975). Gerontological data of C57BL/6J mice. I. Sex differences in survival curves. J. Gerontol..

[29] https://insights.envigo.com/hubfs/resources/data-sheets/2018s-datasheet-0915.pdf.

[30] Schneider C.A., Rasband W.S., Eliceiri K.W. (2012). NIH Image to ImageJ: 25 years of image analysis. Nat. Methods.

[31] Taylor S.C., Berkelman T., Yadav G., Hammond M. (2013). A defined methodology for reliable quantification of Western blot data. Mol. Biotechnol..

[32] Taylor S.C., Posch A. (2014). The design of a quantitative western blot experiment. Biomed. Res. Int..

[33] Ghanemi A., Melouane A., Mucunguzi O., Yoshioka M., St-Amand J. (2018). Energy and metabolic pathways in trefoil factor family member 2 (Tff2) KO mice beyond the protection from high-fat diet-induced obesity. Life Sci..

[34] Ryu S., Spadaro O., Sidorov S., Lee A.H., Caprio S., Morrison C., Smith S.R., Ravussin E., Shchukina I., Artyomov M.N. (2023). Reduction of SPARC protects mice against NLRP3 inflammasome activation and obesity. J. Clin. Investig..

[35] Aoi W., Naito Y., Takagi T., Tanimura Y., Takanami Y., Kawai Y., Sakuma K., Hang L.P., Mizushima K., Hirai Y. (2013). A novel myokine, secreted protein acidic and rich in cysteine (SPARC), suppresses colon tumorigenesis via regular exercise. Gut.

[36] Aoi W., Sakuma K. (2013). Skeletal muscle: Novel and intriguing characteristics as a secretory organ. BioDiscovery.

[37] Usach I., Martinez R., Festini T., Peris J.E. (2019). Subcutaneous Injection of Drugs: Literature Review of Factors Influencing Pain Sensation at the Injection Site. Adv. Ther..

[38] Mathaes R., Koulov A., Joerg S., Mahler H.C. (2016). Subcutaneous Injection Volume of Biopharmaceuticals-Pushing the Boundaries. J. Pharm. Sci..

[39] Shah O.J., Anthony J.C., Kimball S.R., Jefferson L.S. (2000). 4E-BP1 and S6K1: Translational integration sites for nutritional and hormonal information in muscle. Am. J. Physiol. Endocrinol. Metab..

[40] Greiwe J.S., Kwon G., McDaniel M.L., Semenkovich C.F. (2001). Leucine and insulin activate p70 S6 kinase through different pathways in human skeletal muscle. Am. J. Physiol. Endocrinol. Metab..

[41] Barclay R.D., Burd N.A., Tyler C., Tillin N.A., Mackenzie R.W. (2019). The Role of the IGF-1 Signaling Cascade in Muscle Protein Synthesis and Anabolic Resistance in Aging Skeletal Muscle. Front. Nutr..

[42] Anthony J.C., Anthony T.G., Kimball S.R., Jefferson L.S. (2001). Signaling pathways involved in translational control of protein synthesis in skeletal muscle by leucine. J. Nutr..

[43] Kimball S.R., Jefferson L.S., Fadden P., Haystead T.A., Lawrence J.C. (1996). Insulin and diabetes cause reciprocal changes in the association of eIF-4E and PHAS-I in rat skeletal muscle. Am. J. Physiol..

[44] Brunn G.J., Hudson C.C., Sekulic A., Williams J.M., Hosoi H., Houghton P.J., Lawrence J.C., Abraham R.T. (1997). Phosphorylation of the translational repressor PHAS-I by the mammalian target of rapamycin. Science.

[45] Xu G., Marshall C.A., Lin T.A., Kwon G., Munivenkatappa R.B., Hill J.R., Lawrence J.C., McDaniel M.L. (1998). Insulin mediates glucose-stimulated phosphorylation of PHAS-I by pancreatic beta cells. An insulin-receptor mechanism for autoregulation of protein synthesis by translation. J. Biol. Chem..

[46] Kimball S.R., Shantz L.M., Horetsky R.L., Jefferson L.S. (1999). Leucine regulates translation of specific mRNAs in L6 myoblasts through mTOR-mediated changes in availability of eIF4E and phosphorylation of ribosomal protein S6. J. Biol. Chem..

[47] Long W., Saffer L., Wei L., Barrett E.J. (2000). Amino acids regulate skeletal muscle PHAS-I and p70 S6-kinase phosphorylation independently of insulin. Am. J. Physiol. Endocrinol. Metab..

[48] Lira V.A., Benton C.R., Yan Z., Bonen A. (2010). PGC-1alpha regulation by exercise training and its influences on muscle function and insulin sensitivity. Am. J. Physiol. Endocrinol. Metab..

[49] Winder W.W., Hardie D.G. (1996). Inactivation of acetyl-CoA carboxylase and activation of AMP-activated protein kinase in muscle during exercise. Am. J. Physiol..

[50] Wu Z., Puigserver P., Andersson U., Zhang C., Adelmant G., Mootha V., Troy A., Cinti S., Lowell B., Scarpulla R.C. (1999). Mechanisms controlling mitochondrial biogenesis and respiration through the thermogenic coactivator PGC-1. Cell.

[51] Tando T., Hirayama A., Furukawa M., Sato Y., Kobayashi T., Funayama A., Kanaji A., Hao W., Watanabe R., Morita M. (2016). Smad2/3 Proteins Are Required for Immobilization-induced Skeletal Muscle Atrophy. J. Biol. Chem..

[52] Goodman C.A., McNally R.M., Hoffmann F.M., Hornberger T.A. (2013). Smad3 induces atrogin-1, inhibits mTOR and protein synthesis, and promotes muscle atrophy in vivo. Mol. Endocrinol..

[53] Nishimura Y., Chunthorng-Orn J., Lord S., Musa I., Dawson P., Holm L., Lai Y.C. (2022). Ubiquitin E3 ligase Atrogin-1 protein is regulated via the rapamycin-sensitive mTOR-S6K1 signaling pathway in C2C12 muscle cells. Am. J. Physiol. Cell Physiol..

[54] Schiaffino S., Mammucari C. (2011). Regulation of skeletal muscle growth by the IGF1-Akt/PKB pathway: Insights from genetic models. Skelet. Muscle.

[55] Nie J., Sage E.H. (2009). SPARC inhibits adipogenesis by its enhancement of beta-catenin signaling. J. Biol. Chem..

[56] Konigshoff M., Balsara N., Pfaff E.M., Kramer M., Chrobak I., Seeger W., Eickelberg O. (2008). Functional Wnt signaling is increased in idiopathic pulmonary fibrosis. PLoS ONE.

[57] Fan J., Zhang X., Jiang Y., Chen L., Sheng M., Chen Y. (2021). SPARC knockdown attenuated TGF-beta1-induced fibrotic effects through Smad2/3 pathways in human pterygium fibroblasts. Arch. Biochem. Biophys..

[58] Verrecchia F., Mauviel A. (2002). Transforming growth factor-beta signaling through the Smad pathway: Role in extracellular matrix gene expression and regulation. J. Investig. Dermatol..

[59] Rivera L.B., Brekken R.A. (2011). SPARC promotes pericyte recruitment via inhibition of endoglin-dependent TGF-beta1 activity. J. Cell Biol..

[60] Nakamura K., Nakano S., Miyoshi T., Yamanouchi K., Nishihara M. (2013). Loss of SPARC in mouse skeletal muscle causes myofiber atrophy. Muscle Nerve.

[61] Song H., Guan Y., Zhang L., Li K., Dong C. (2010). SPARC interacts with AMPK and regulates GLUT4 expression. Biochem. Biophys. Res. Commun..

[62] Theeuwes W.F., Gosker H.R., Langen R.C.J., Pansters N.A.M., Schols A., Remels A.H.V. (2018). Inactivation of glycogen synthase kinase 3beta (GSK-3beta) enhances mitochondrial biogenesis during myogenesis. Biochim. Biophys. Acta Mol. Basis Dis..

[63] Gingras A.C., Gygi S.P., Raught B., Polakiewicz R.D., Abraham R.T., Hoekstra M.F., Aebersold R., Sonenberg N. (1999). Regulation of 4E-BP1 phosphorylation: A novel two-step mechanism. Genes. Dev..

[64] Flores-Opazo M., McGee S.L., Hargreaves M. (2020). Exercise and GLUT4. Exerc. Sport Sci. Rev..

[65] Buford T.W., Cooke M.B., Willoughby D.S. (2009). Resistance exercise-induced changes of inflammatory gene expression within human skeletal muscle. Eur. J. Appl. Physiol..

[66] Jung S., Kim K. (2014). Exercise-induced PGC-1α transcriptional factors in skeletal muscle. Integr. Med. Res..

[67] Mavropalias G., Wu Y.F., Boppart M.D., Blazevich A.J., Nosaka K. (2022). Increases in Integrin-ILK-RICTOR-Akt Proteins, Muscle Mass, and Strength after Eccentric Cycling Training. Med. Sci. Sports Exerc..

[68] Chen M.J., Russo-Neustadt A.A. (2005). Exercise activates the phosphatidylinositol 3-kinase pathway. Brain Res. Mol. Brain Res..

[69] Yang Y., Creer A., Jemiolo B., Trappe S. (2005). Time course of myogenic and metabolic gene expression in response to acute exercise in human skeletal muscle. J. Appl. Physiol..

[70] Nikooie R., Jafari-Sardoie S., Sheibani V., Nejadvaziri Chatroudi A. (2020). Resistance training-induced muscle hypertrophy is mediated by TGF-β1-Smad signaling pathway in male Wistar rats. J. Cell Physiol..

[71] Aoi W., Hirano N., Lassiter D.G., Björnholm M., Chibalin A.V., Sakuma K., Tanimura Y., Mizushima K., Takagi T., Naito Y. (2019). Secreted protein acidic and rich in cysteine (SPARC) improves glucose tolerance via AMP-activated protein kinase activation. FASEB J. Off. Publ. Fed. Am. Soc. Exp. Biol..

[72] Hoffmann C., Weigert C. (2017). Skeletal Muscle as an Endocrine Organ: The Role of Myokines in Exercise Adaptations. Cold Spring Harb. Perspect. Med..

[73] Gomarasca M., Banfi G., Lombardi G. (2020). Myokines: The endocrine coupling of skeletal muscle and bone. Adv. Clin. Chem..

[74] Severinsen M.C.K., Pedersen B.K. (2020). Muscle-Organ Crosstalk: The Emerging Roles of Myokines. Endocr. Rev..

[75] Garneau L., Parsons S.A., Smith S.R., Mulvihill E.E., Sparks L.M., Aguer C. (2020). Plasma Myokine Concentrations After Acute Exercise in Non-obese and Obese Sedentary Women. Front. Physiol..

[76] Frontera W.R., Hughes V.A., Fielding R.A., Fiatarone M.A., Evans W.J., Roubenoff R. (2000). Aging of skeletal muscle: A 12-yr longitudinal study. J. Appl. Physiol..

[77] Yamada M., Moriguch Y., Mitani T., Aoyama T., Arai H. (2014). Age-dependent changes in skeletal muscle mass and visceral fat area in Japanese adults from 40 to 79 years-of-age. Geriatr. Gerontol. Int..

[78] Miyamoto T., Shimizu Y., Matsuo Y., Otaru T., Kanzawa Y., Miyamae N., Yamada E., Katsuno T. (2021). Effects of exercise intensity and duration on a myokine, secreted protein acidic and rich in cysteine. Eur. J. Sport Sci..

