# Supplementary material for: A Single Intraperitoneal Secreted Protein Acidic and Rich in Cysteine Injection in Mice Is Towards an Exercise-like Phenotype
Source: Biology (Basel). 2025 Apr 10;14(4):398. doi: 10.3390/biology14040398 (PMC12025124; doi:10.3390/biology14040398)

Appendix 3: Total proteins after the transfer and target protein blots

ILK

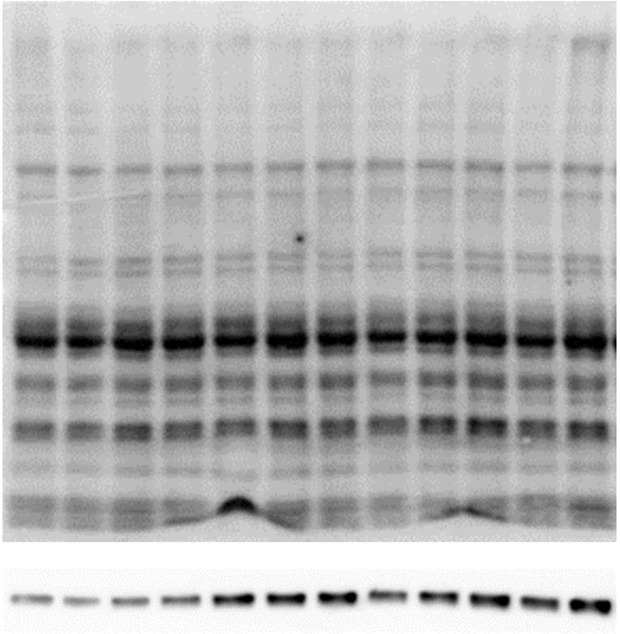

pGSK3β

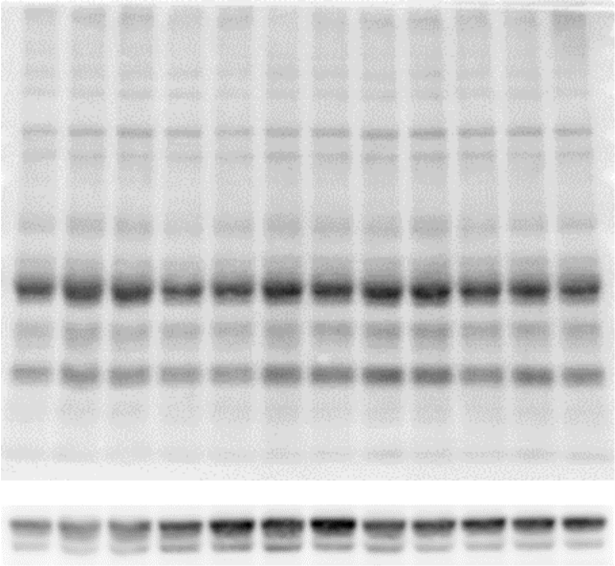

GSK3β

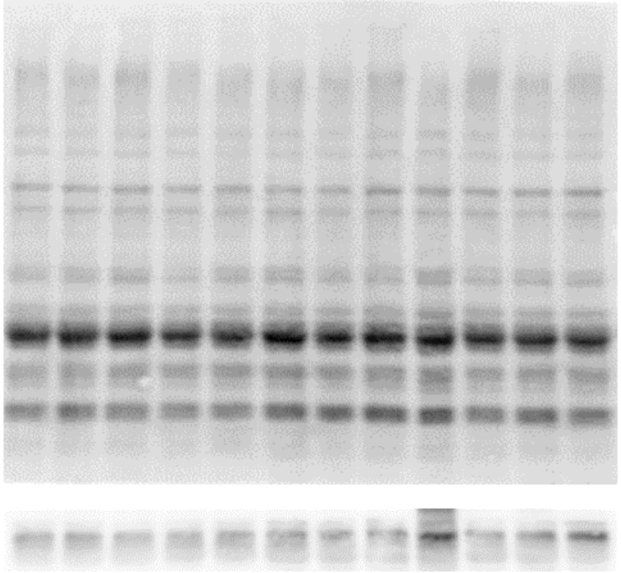

COL1A2

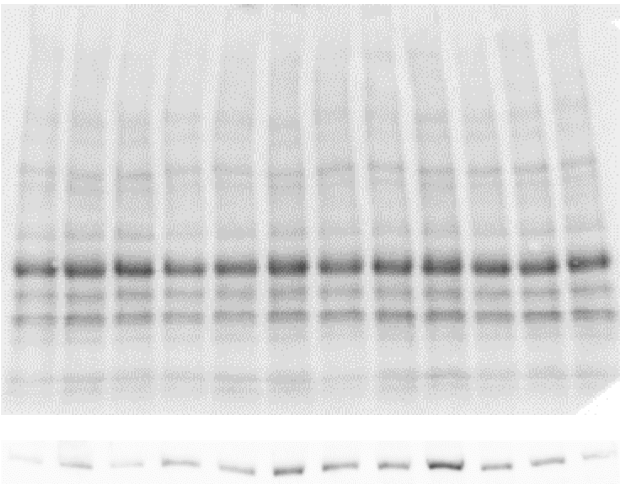

pAkt1

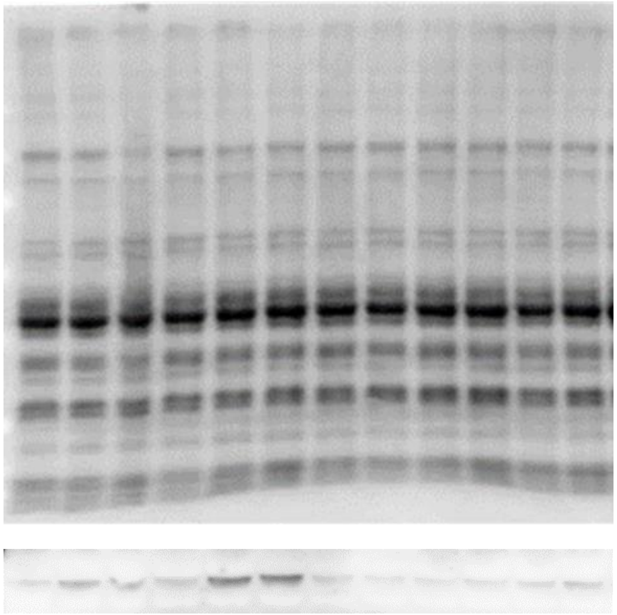

pS6K

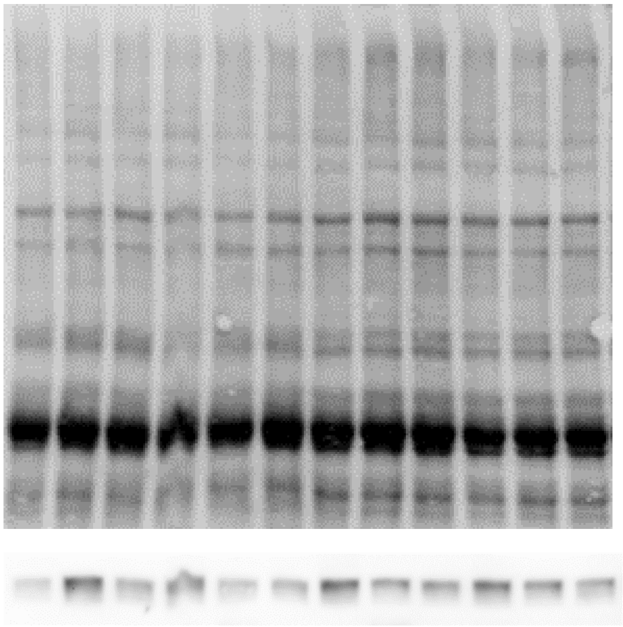

p4EBP1

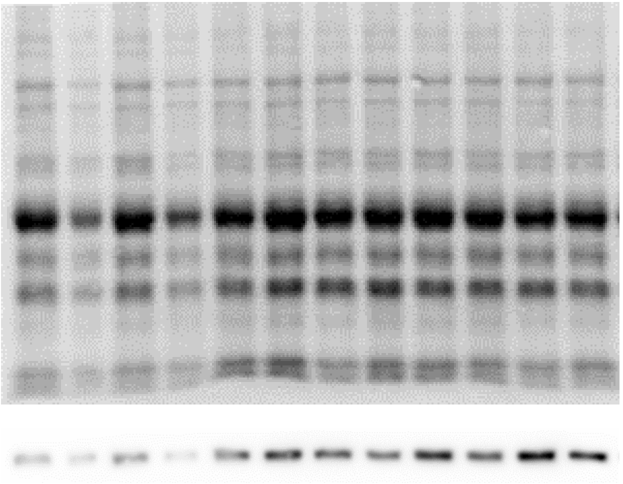

4EBP1

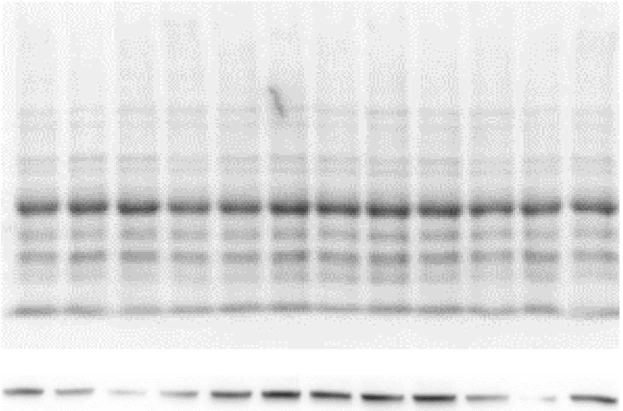

IL6

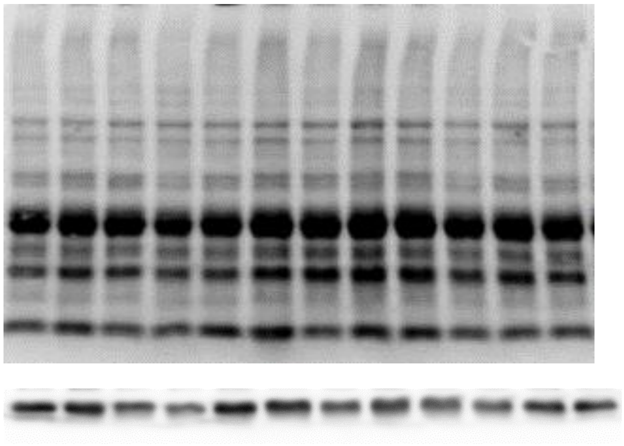

pAMPK

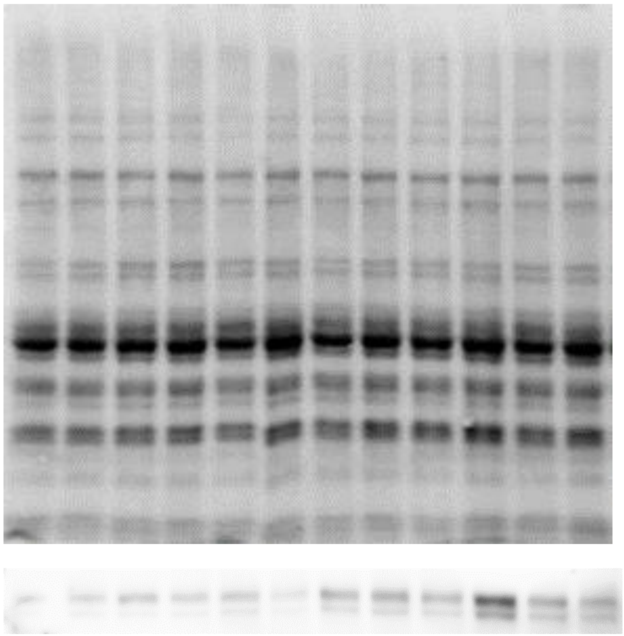

PGC1α

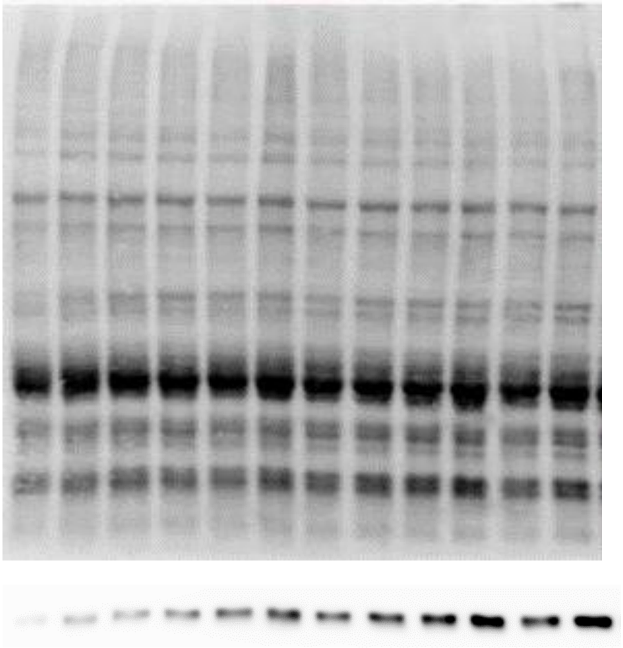

GLUT4

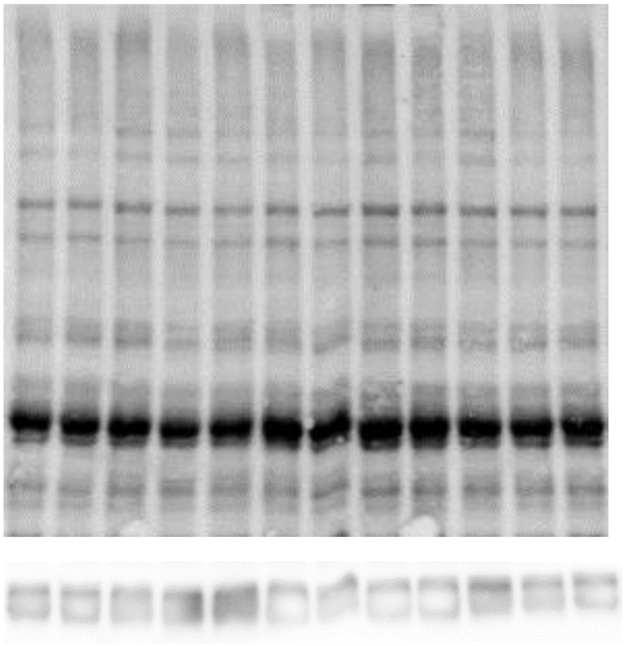

NRF1

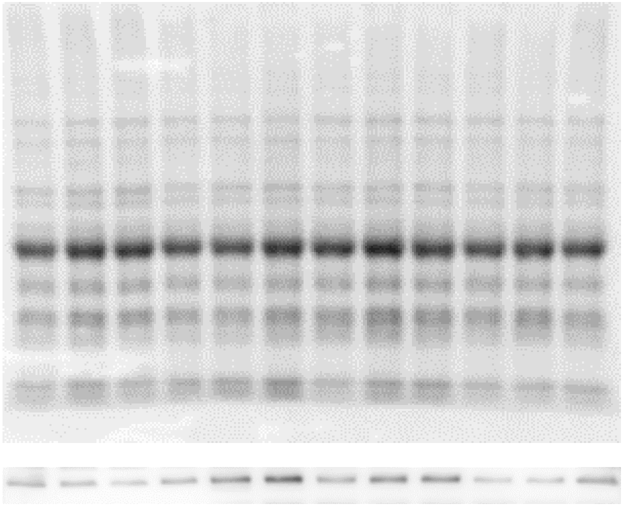

COX2

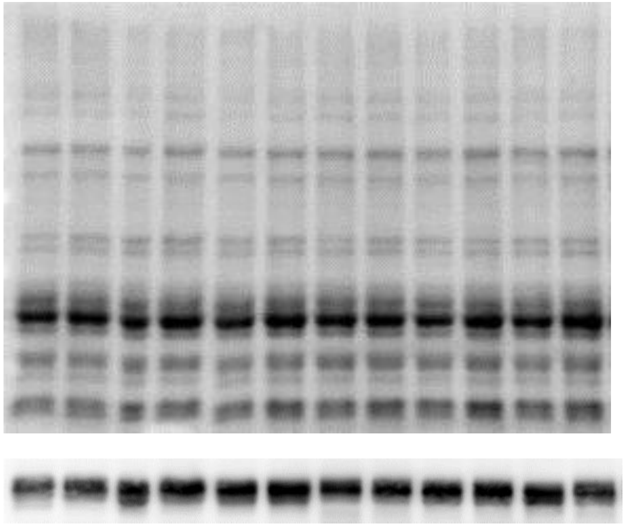

pSmad3

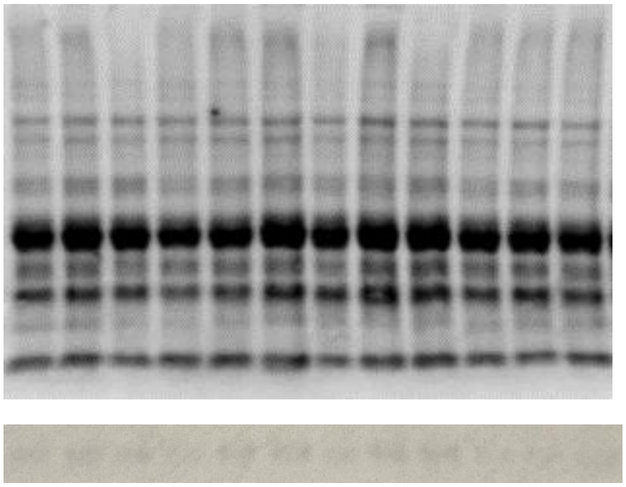

SDHB

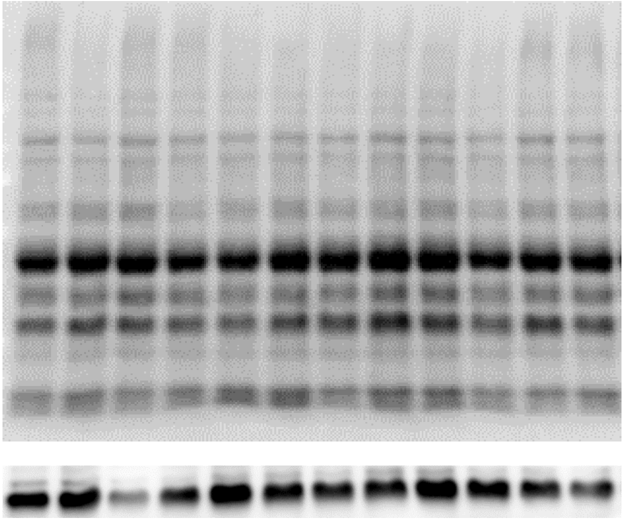

pFOXO1

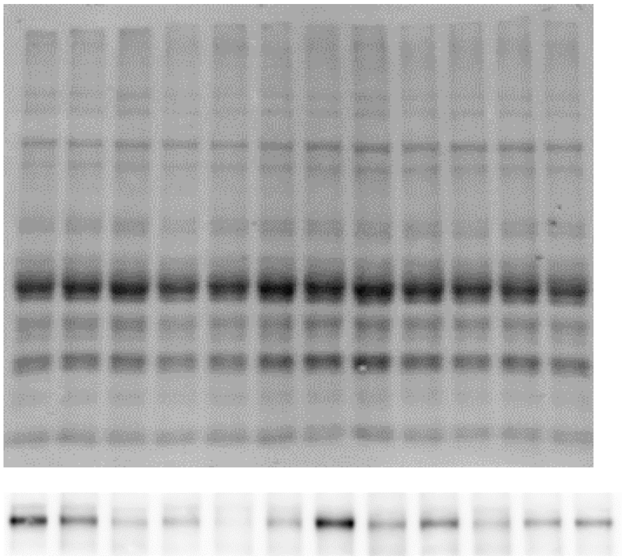

TRIM63

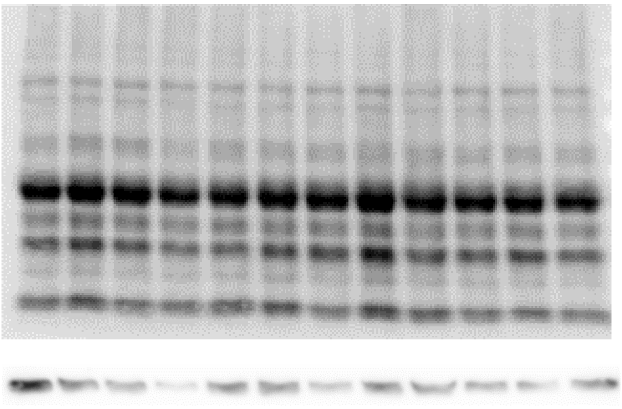

FBX32

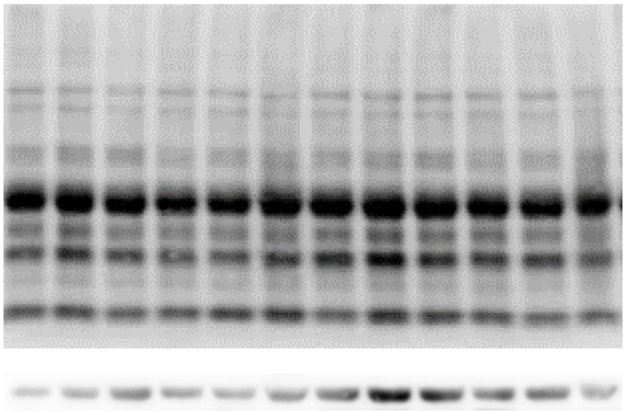

Myod1

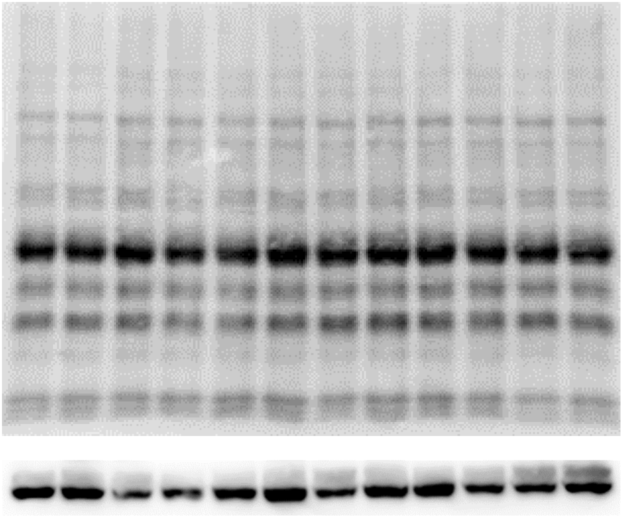

Myogenin

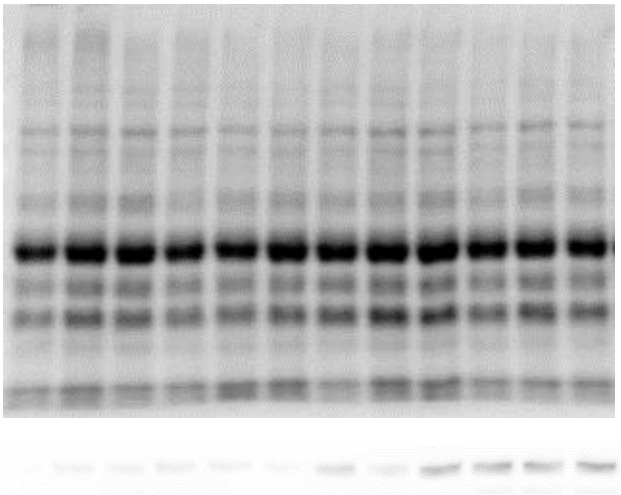

Supplement: Supplementary file 1 [file biology-14-00398-s001.zip › Supplementary S3_biology-3520992.pdf]
