# Supplementary material for: A Single Intraperitoneal Secreted Protein Acidic and Rich in Cysteine Injection in Mice Is Towards an Exercise-like Phenotype
Source: Biology (Basel). 2025 Apr 10;14(4):398. doi: 10.3390/biology14040398 (PMC12025124; doi:10.3390/biology14040398)
Supplement: Supplementary file 1 [file biology-14-00398-s001.zip › Supplementary S2_biology-3520992.pptx]

## Slide 1
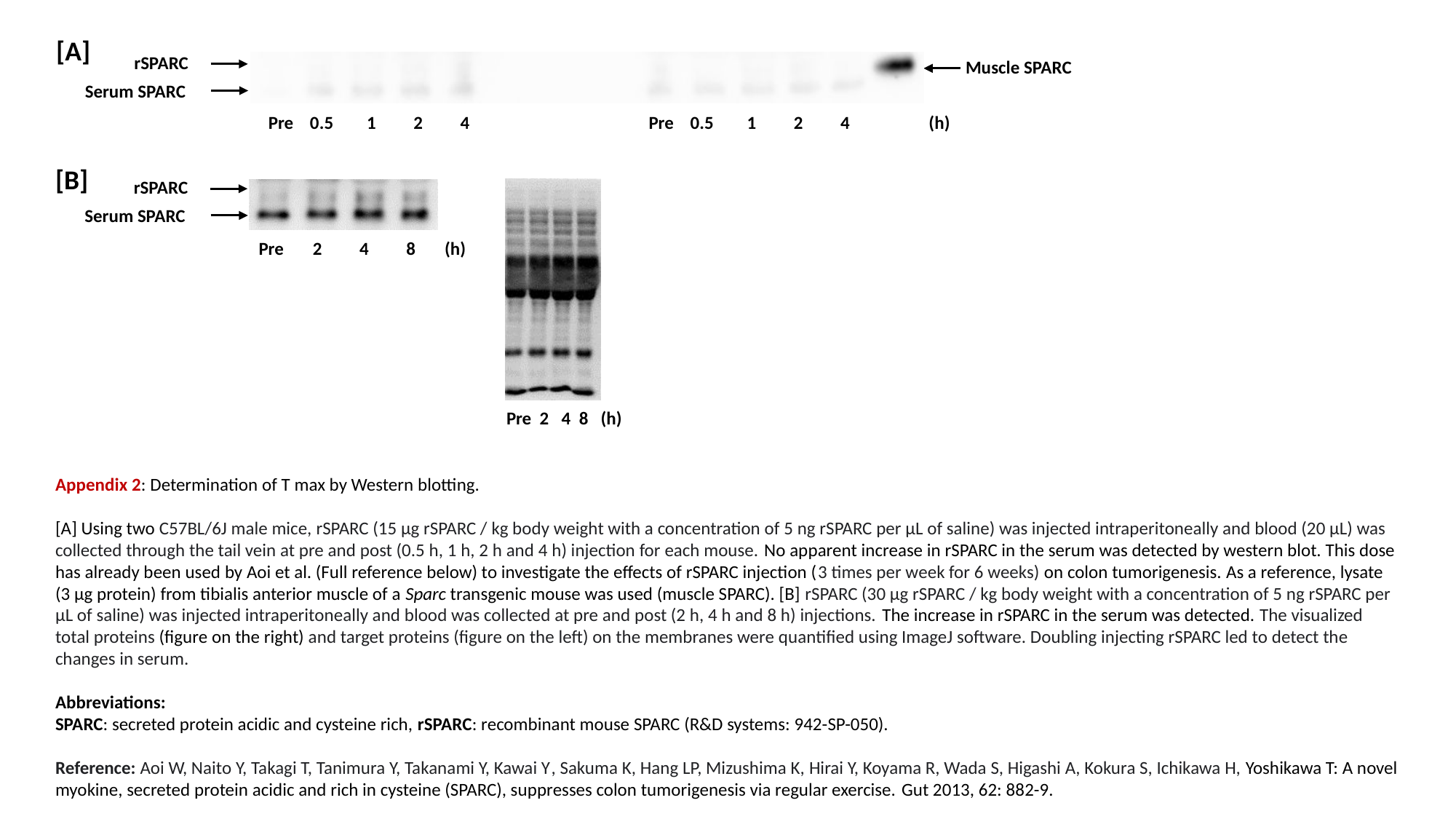

[A]
rSPARC
Serum SPARC
Muscle SPARC
 Pre 0.5 1 2 4 Pre 0.5 1 2 4 (h)
[B]
rSPARC
Serum SPARC
Pre 2 4 8 (h)
 Pre 2 4 8 (h)
Appendix 2: Determination of T max by Western blotting.
[A] Using two C57BL/6J male mice, rSPARC (15 µg rSPARC / kg body weight with a concentration of 5 ng rSPARC per µL of saline) was injected intraperitoneally and blood (20 µL) was collected through the tail vein at pre and post (0.5 h, 1 h, 2 h and 4 h) injection for each mouse. No apparent increase in rSPARC in the serum was detected by western blot. This dose has already been used by Aoi et al. (Full reference below) to investigate the effects of rSPARC injection (3 times per week for 6 weeks) on colon tumorigenesis. As a reference, lysate (3 µg protein) from tibialis anterior muscle of a Sparc transgenic mouse was used (muscle SPARC). [B] rSPARC (30 µg rSPARC / kg body weight with a concentration of 5 ng rSPARC per µL of saline) was injected intraperitoneally and blood was collected at pre and post (2 h, 4 h and 8 h) injections. The increase in rSPARC in the serum was detected. The visualized total proteins (figure on the right) and target proteins (figure on the left) on the membranes were quantified using ImageJ software. Doubling injecting rSPARC led to detect the changes in serum.
Abbreviations:
SPARC: secreted protein acidic and cysteine rich, rSPARC: recombinant mouse SPARC (R&D systems: 942-SP-050).
Reference: Aoi W, Naito Y, Takagi T, Tanimura Y, Takanami Y, Kawai Y, Sakuma K, Hang LP, Mizushima K, Hirai Y, Koyama R, Wada S, Higashi A, Kokura S, Ichikawa H, Yoshikawa T: A novel myokine, secreted protein acidic and rich in cysteine (SPARC), suppresses colon tumorigenesis via regular exercise. Gut 2013, 62: 882-9.
